# Freeze-Dried Cooked Chickpeas: Considering a Suitable Alternative to Prepare Tasty Reconstituted Dishes

**DOI:** 10.3390/foods12122339

**Published:** 2023-06-10

**Authors:** M. Isabel Cambero, Gonzalo Doroteo García de Fernando, M. Dolores Romero de Ávila, Víctor Remiro, Luis Capelo, José Segura

**Affiliations:** Department of Food Technology, Faculty of Veterinary Medicine, Complutense University of Madrid, Av. Puerta de Hierro s/n., 28040 Madrid, Spain; icambero@ucm.es (M.I.C.); mingui@ucm.es (G.D.G.d.F.); mdavilah@ucm.es (M.D.R.d.Á.); vremiro@ucm.es (V.R.);

**Keywords:** chickpea, freeze-drying, rehydration, instant meal, *Cocido*, *easy-to-prepare*, reconstituted meal

## Abstract

The current trend in food consumption is toward convenience, i.e., fast food. The present work aims to study the potential of incorporating freeze-dried cooked chickpeas into a complex and traditional dish in Spanish gastronomy, such as *Cocido*, which has this legume as the main ingredient. *Cocido* is a two-course meal: a thin-noodle soup and a mix of chickpeas, several vegetables, and meat portions. The textural properties, sensory qualities, and rehydration kinetics of chickpeas of three Spanish varieties were investigated to select the most suitable cooking conditions to obtain freeze-dried chickpeas of easy rehydration whilst maintaining an adequate sensory quality for the preparation of the traditional dish. The sensory quality of various vegetables and meat portions, cooked under different conditions, was evaluated after freeze-drying and rehydration. It was possible to reproduce the sensory quality of the traditional dish after rehydration with water, heating to boiling in a microwave oven for 5 min, and resting for 10 min. Therefore, it is possible to commercialize complex dishes based on pulses and other cooked and freeze-dried ingredients as reconstituted meals with a wide nutrient profile. Nevertheless, additional research is required on the shelf life, together with other economic and marketing issues such as design of a proper packaging, that would allow consumption as a two-course meal.

## 1. Introduction

Today, consumer lifestyles require foods that can be prepared quickly. More than ever, healthy, nutritional, and appetizing “instant/reconstituted meals”, meaning convenience meals, are sought out by consumers. Instant or reconstituted meals (“*easy-to-prepare*”) are characterized by minimal processing before eating. Mostly, these products are constituted by a dehydrated base (soup, vegetables, pasta, etc.) and consumed in the container itself after rehydration with boiling water [1].

In the current market, reconstituted meals based on instant noodles are widespread. For decades, dried noodles and instant fried noodles have been the most consumed products due to their ease of preservation and cooking [2]. Nevertheless, the offer of a reconstituted meal based on a complex dish is almost non-existent. Research is being carried out to increase both the quality and the variety of such products [2,3,4]. Chen et al. [5] observed that the consumption of dry solid ingredients in instant soups in the modern era is increasing rapidly. Therefore, the authors reviewed the recent changes in the quality of the solid ingredients of instant soups. Pieniazek and Messina [6] analyzed the microstructure and texture of legumes and other vegetables for instant meals using scanning electron microscopy combined with an image processing technique. Anecdotally, even America’s space agency, NASA (National Aeronautics and Space Administration) is interested in the preservation of vitamins, a low weight/volume ratio in packaging, and the retention of the sensory quality of the instant/reconstituted meals [7].

In this line of work, freeze-drying is one of the ideal drying methods for the preservation of foods that are susceptible to alterations due to heat and/or oxidation. Freeze-dried products have low moisture content and usually a porous structure and can be reconstituted with a high rehydration speed [8,9]. The freeze-drying method allows food to preserve its original color, flavor, aroma, and appearance to the maximum degree possible while protecting its composition [10]. Nutritional value is not altered since protein denaturation and vitamin loss do not occur [11].

Chickpeas are the most consumed leguminous crop in many countries around the world, including Spain. They are characterized by having a low amount of lipids and a significant amount of all the essential amino acids, except sulfur-containing ones, followed by tryptophan and threonine [12,13].

Nevertheless, from a culinary point of view, they are usually combined with other ingredients due to their mild flavor [14].

The chickpea is a legume that can be used together with other vegetables and different types of meat to fulfill the nutrients of a balanced diet [15]. An example of such a combination is a traditional Spanish dish known as *Cocido*, of which there are different varieties, such as *Cocido Madrileño*, *Cocido Extremeño*, *Cocido Andaluz*, *Cocido Maragato*, etc. All these dishes have chickpeas in common as the main ingredient. This pulse is cooked together with different types of vegetables and meat (beef, chicken, and pork). *Cocido Madrileño* is usually eaten in two or three courses: once the chickpeas, meat portions, and vegetables are cooked, the broth is separated and used to make a soup with thin noodles, which is served as the first course. The remaining ingredients are then made into the second course. Often, the chickpeas and vegetables are eaten first, followed by meat pieces [16,17].

This work studied the effect of cooking conditions on the rehydration kinetics of different varieties of cooked and freeze-dried chickpeas. In addition, the effect of cooking on the textural attributes and sensory characteristics of rehydrated chickpeas was studied. Conversely, the sensory attributes of different vegetables and portions of meat of different species, freeze-dried after cooking and subsequent rehydration, were analyzed. The final objective of this work was to determine the potential use of chickpeas, vegetables and meat portions, freeze-dried after cooking, for the assembly of a complex dish such as *Cocido Madrileño* for its consumption as an *easy-to-prepare* dish, which only requires rehydration and heating for its consumption. The aim is to offer an alternative to bring traditional gastronomy closer to the preparation of *easy-to-prepare* food.

## 2. Materials and Methods

### 2.1. Ingredients (Raw Products)

Regarding chickpea (*Cicer arietinum* L.), the three most known varieties in Spain were considered: *Pedrosillano* (PD, ecotype guarantee mark from *Pedrosillo el Ralo*, in the region of *La Armuña*, Salamanca, Spain), *Castellano* (CA, *La Moraña*, Ávila, Spain), and *Blanco Lechoso* (BL, *Marchena*, Sevilla, Spain). In agreement with the literature, PD showed a caliber range of 320–360 beans/100 g, whereas CA and BL showed 190–210 beans/100 g [18,19,20,21].

**Figure 1:**
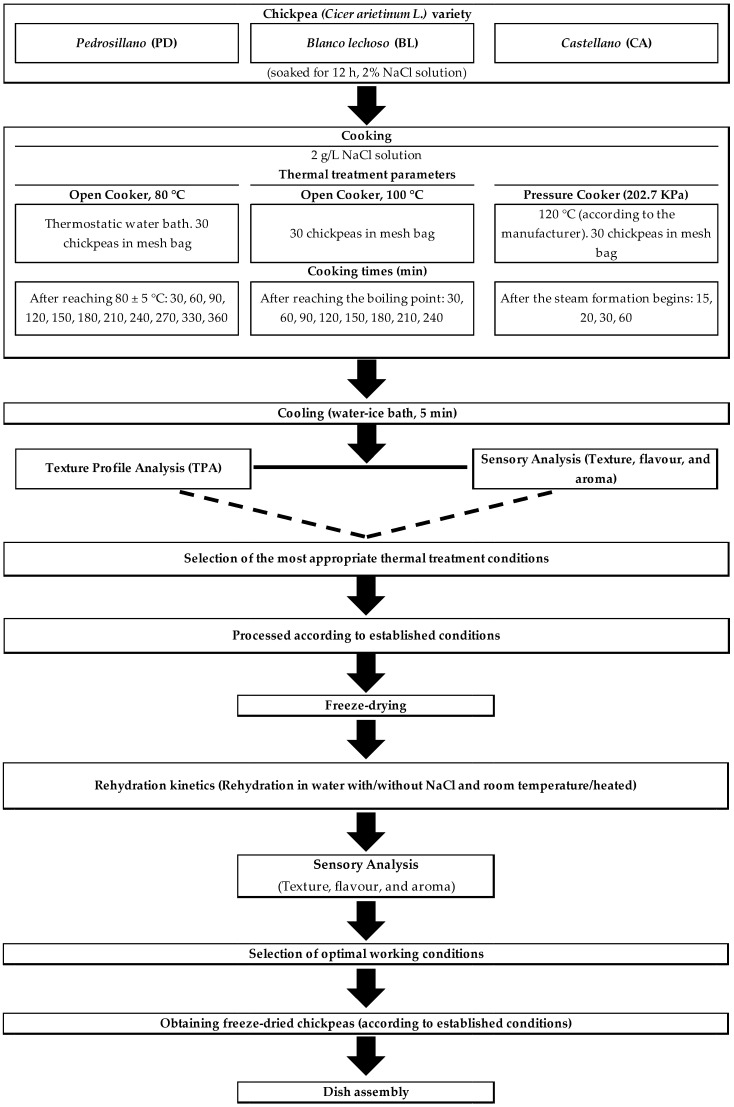
Process followed for the selection of the preparation conditions of different Spanish varieties of chickpeas (*Cicer arietinum*).

**Figure 2:**
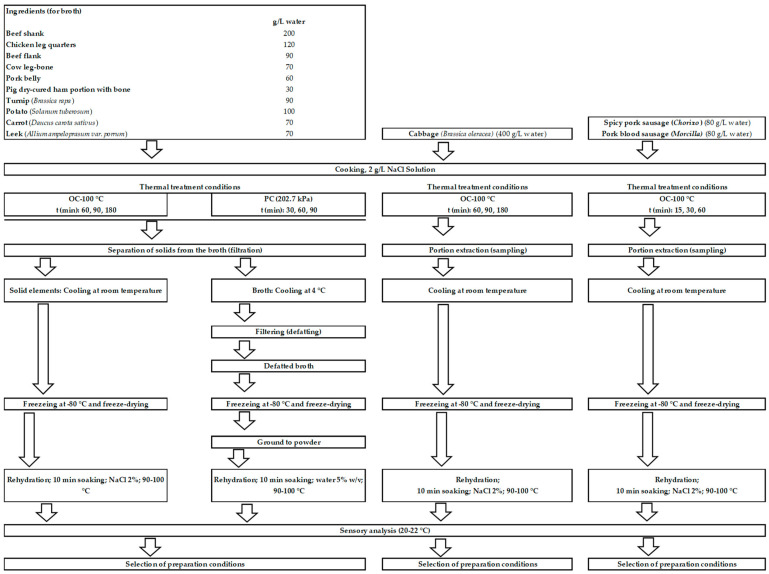
Process followed for the selection of the preparation conditions of different ingredients (meat and vegetables) of the dish. OC = open cooker; PC = pressure cooker.

The procedure followed in this study is summarized in Figure 1. According to traditional *Cocido* recipes [16,17], the following ingredients, together with chickpeas, were used (Figure 2): beef shank and flank, chicken leg-quarters, cow leg-bone, pork belly, pig dry-cured ham bone, *chorizo* (spicy pork sausage), *morcilla* (pork blood sausage), turnip (*Brassica napus*), potato (*Solanum tuberosum*), carrot (*Daucus carota sativus*), leek (*Allium ampeloprasum var. porrum*), and cabbage (*Brassica oleracea*). Meat products were obtained from a local butcher shop and vegetables from a local greengrocer (Madrid, Spain).

### 2.2. Cooking Conditions

#### 2.2.1. Chickpea Processing

The chickpeas were soaked in a 2% NaCl aqueous solution for 12 h and rinsed with fresh water before the thermal treatment. Afterward, mesh bags with 30 chickpeas were prepared to be extracted from the cooker at each evaluated temperature and time: Open cooker (OC, atmospheric pressure): temperature of 80 °C with times of 30 (OC-30), 60 (OC-60), 90 (OC-90), 120 (OC-120), 150 (OC-150), 180 (OC-180), 210 (OC-210), and 240 (OC-240) min; and temperature of 100 °C with the same times. Pressure cooker (PC, 202.7 KPa, 120 °C, according to manufacturer) with times of 15 (PC-15), 20 (PC-20), 30 (PC-30) and 60 (PC-60) min. In all cases, after cooking, the mesh bags were submerged for 5 min in an ice-water solution to stop the cooking process. Each group was split into three portions: two were prepared for textural and sensorial analyses, and the third was frozen and freeze-dried (see below).

#### 2.2.2. Processing of Meat and Vegetables as well as Broth Preparation

Both OC and PC processed/cooked, with 1 L of distilled water with 2 g NaCl, about 200 g beef shank, 120 g chicken leg-quarters, 90 g beef flank, 70 g cow leg-bone, 60 g pork belly, 30 g pig dry-cured ham portion with bone, 90 g turnip (*Brassica napus*), 100 g potato (*Solanum tuberosum*), 70 g carrot (*Daucus carota sativus*) and 70 g leek (*Allium ampeloprasum var. porrum*). After the designated cooking times, the ingredients were quickly extracted, separated, and cooled to room temperature (20–22 °C) and then packed in plastic bags to be frozen for freeze-drying. The broth was cooled to 4 °C and filtered (1 mm mesh) to separate the solidified fat. The defatted broth was also packed in plastic bags and frozen for subsequent freeze-drying (Figure 2).

In addition, cabbage (*Brassica oleracea*) was cooked alone (400 g/L of distilled water with 2 g of NaCl, for 60, 90, and 180 min). Spicy pork sausage (*Chorizo*) and pork blood sausage (*Morcilla*) were cooked together but separately from the other ingredients (80 g of each meat product/L distilled water, no NaCl, for 15, 30, and 60 min) (Figure 2). After the cooking times, these products were extracted, and they were cooled and frozen using the same method as indicated above. In these cases, the cooking water was discarded. Ten repetitions were carried out for each cooking condition, processing the amounts corresponding to 4 L of distilled water in each of them.

### 2.3. Texture Profile Analysis of Chickpeas

Texture profile analysis (TPA) was carried out using a TA.XT2i SMS Stable Micro Systems Texture Analyzer (Stable Microsystems Ltd., Surrey, England) with the Texture Expert program. Textural tests were carried out at 20–22 °C. A double compression cycle test was performed up to 50% compression of the original portion height with an aluminum cylinder probe of 2 cm diameter (5 s were allowed to elapse between two compression cycles). Force–time deformation curves were obtained with a 50-kg load cell applied at a crosshead speed of 2 mm/s. Hardness (the maximum force required to compress the sample, N), springiness (the ability of the sample to recover its original form after deforming force was removed, m), adhesiveness (the area under the abscissa after the first compression, N×s) and cohesiveness (the extent to which the sample could be deformed before rupture) were quantified according to Segura et al. [22].

### 2.4. Freeze-Drying and Rehydration Studies

Frozen samples (chickpeas, broth, and the other vegetable and meat ingredients) at −80 °C were freeze-dried under vacuum in a Lyoquest Lyophilizer (Telstar, Terrasa, Spain) with a chamber pressure of 0.03 bar, −70 °C condenser temperature, and 20 °C shelf temperature. During sublimation drying, the temperature was kept below eutectic. In the desorption drying, the temperature was kept below 24 °C.

Freeze-dried samples were placed inside a glass container and vacuum-packed (20 kPa) in plastic bags (10 cm^2^; laminated film: polyamide and polyethylene; thickness of 90 µm; low gas permeability: O_2_ and CO_2_ transmission rates of 35 cm^3^/24 h m^2^ bar) to avoid any damage to the structure of the solid ingredients, and stored at −20 °C.

Chickpeas were rehydrated by soaking in two different media: distilled water and 2% (*w*/*v*) NaCl solution. In both cases, the studies were carried out at room (20–22 °C) and close to boiling (90–100 °C) temperatures. The chickpea rehydration kinetics were developed by weighing them every 2 min for 25 min (the time at which no statistically significant differences (*p* > 0.05) were detected between consecutive measurements). At specific times, 10 chickpeas were extracted and blotted free of excess surface moisture by being placed separately on a stainless-steel mesh (1 mm hole diameter) with a Reshma paper filter at room temperature for 3 min and then weighed. For rehydration of the broth, the substrate obtained after freeze-drying was reduced to a powder using a mortar. This operation was performed immediately before rehydration using distilled hot water (90–100 °C). The ratio of powder to water was 2.5% (*w*/*v*). Rehydration of the other cooked ingredients was performed with 2% (*w*/*v*) NaCl water solution (90–100 °C) or in the hot rehydrated broth.

### 2.5. Sensory Evaluation

Eight semi-trained panelists (four females and four males) with knowledge of the product were asked to carry out a rank order test [23,24] in a taste panel area equipped with individual booths according to the methodology guidelines for sensory analysis from the International Standards Organization 6658:2017 [25]. Panelists analyzed the chickpeas, the vegetables, and the meat ingredients separately, as well as the broth, after different cooking and rehydration procedures. The samples were maintained in a water bath at 35 ± 5 °C for no more than 1 h. For the sensory evaluation of these products, the panelists were instructed to rank samples by their liking/satisfaction level considering the texture, the taste/flavor, and the aroma, by taking as reference the expected characteristics in a whole *Cocido* dish or each of its ingredients individually. Each sample was given a different score; the sample with the least appropriate sensory characteristics was allocated a score of 1, and the sample with the most satisfactory sensory characteristics and close to the *Cocido* dish was assigned the highest score, which coincided with the number of samples tasted. To obtain the final score of each sample, the sum of ranks was calculated corresponding to the sum of scores of the sample sensory characteristics. This was estimated by calculating the sum of the products of the values given to each sample by the number of times each sample was allocated to a specific score: (1 × n_1_) + (2 × n_2_) + (3 × n_3_) + … + (*g* × n*_g_*), where n_1_ = the frequency that the sample receives score 1 (worst sensory characteristics), n_2_ = frequency of score 2, etc., and n*_g_* = frequency of the highest score (*g*, best sensory characteristics).

Together with the ranking, panelists were asked to indicate the causes of each score and were offered the possibility to point out possible weaknesses or defects in a “descriptive” section.

The complete culinary preparation was tasted after rehydration using a descriptive test of each sensory attribute (flavor, aroma, texture) of the *Cocido Madrileño* dishes (soup, chickpeas, vegetables, and meats).

### 2.6. Statistical Analysis

The statistical analysis was carried out using Statgraphics Plus version 5.1. Data were brought forward as the mean ± the standard deviation (SD) of each treatment. After determining the goodness of fit of the data to a normal distribution (95% confidence) using the Shapiro–Wilks test, Duncan’s test for multiple mean comparisons was applied to ascertain differences among means. Multifactor ANOVA was conducted to determine the significant effects produced by chickpea variety, cooking time and temperature associated with the culinary treatment (OC and PC), and their combinations, on the textural parameters of chickpeas. Analyses were carried out in triplicate. The significance level (*p* < 0.05) of data obtained in the rank order test was determined by Friedman’s rank addition using the tables for multiple comparison procedures for the analysis of ranked data [26].

## 3. Results and Discussion

### 3.1. Texture Profile and Sensory Analyses of Cooked Chickpeas

The statistical analysis of hardness, adhesiveness, springiness, and cohesiveness of chickpeas, depending on the cooking temperature, time, and chickpea variety, together with the cooking procedure (OC and PC), is shown in Table 1. The multifactor ANOVA results indicated significant interactions (*p* < 0.05) between the effect of cooking conditions (temperature and time) and chickpea variety on hardness, adhesiveness, and springiness. It should be noted that, as customary, before cooking, a 12 h soaking in a NaCl solution was carried out. According to Fabbri et al. [27] and Wood [28], soaking with salt, discarding the water, rinsing, and boiling was the best procedure to remove anti-nutrients and increase iron absorption, reduce cooking time and gastric issues, and improve the protein quality, texture, and appearance of pulses.

For OC-30 at 80 °C, CA chickpeas showed the highest hardness value, followed by BL and PD. Still considering cooking at 80 °C, the highest decrease with time in harness values was detected for BL (89.5%), followed by CA (81.5%) and PD (65.9%). Consequently, at OC-240 at 80 °C, BL showed a lower value of hardness than CA and PD, but no difference was observed between CA and PD (3.64 N and 11.2 N, respectively). When cooking at 100 °C, no statistical differences in hardness values, which were lower than 1 N, were observed after 120 min (0.22 N; *p* > 0.05). When using PC, no statistical differences were detected in hardness (0.18 N; *p* > 0.05) among the chickpea varieties. Nevertheless, cooking time showed a statistical tendency to lower hardness values when the cooking time was longer (*p* = 0.095; 0.19 N vs. 0.17 N; 10.7% difference).

Chickpea seeds are usually cooked above the gelatinization temperature to soften the grain and improve the nutritional quality and aroma development, resulting in the improvement of overall palatability [29,30]. The hydrothermal cooking process of chickpeas involves water absorption through the seed coat, until reaching an equilibrium condition, followed by structural changes in the seed by heat, such as separation of cells, gelatinization of cell starch of the cotyledons, protein denaturation, and deformation of the spherical granules [31]. Starch gelatinization occurs between 60 and 95 °C [32]; however, it will only occur when the cotyledon moisture content is sufficiently high.

The main conventional cooking method for legume seeds is boiling them in water for an extended period in either OC or PC conditions. During PC, heat is evenly, deeply, and quickly distributed, and faster cooking than OC has been described [33]. The higher intensity of the heat treatment in PC may be the reason for not finding significant differences in hardness among the chickpea varieties. However, in OC, the heating at lower temperatures and the slower heating would allow observing the effect of the thermal treatment on the chickpea structures and, consequently, a dependence of hardness on the chickpea variety.

Güzel and Sayar [33] found a higher percentage of seed coat splits in OC than PC, although the amounts of solid lost were higher when the legumes were PC processed. In addition, higher levels of resistant starch and lower levels of slowly digestible starch were detected when PC was used.

No differences in adhesiveness were detected in OC at 80 °C for the different cooking times and chickpea varieties (−0.004 N×s). Nevertheless, higher absolute values were detected when cooking at 100 °C than at 80 °C (77.0% difference in BL and CA but 97.5% in PD; *p* < 0.0001). When cooking at 100 °C, adhesiveness absolute values tended to increase with cooking time. Furthermore, PD showed six times higher absolute values than BL and CA (−0.138 vs. −0.023 N×s, respectively; *p* < 0.0001). In the case of PC, higher (in absolute value) values were detected in adhesiveness directly related to cooking time. In addition, such an increase was higher for PD than for BL and CA (68.2% vs. 42.0% difference, respectively; *p* < 0.0001). Accordingly, Wani et al. [34] described that swelling capacity had a positive correlation with cooking time and water uptake ratio but a negative correlation with cooked seed hardness and adhesiveness.

Related to springiness, a dependence on the cooking temperature was observed; OC at 100 °C showed higher values than OC at 80 °C (18.7% difference; *p* = 0.0401). In addition, CA springiness of OC at 80 °C showed 21.8% higher values than BL and 45.5% higher values than PD, and BL OC at 80 °C values were 30.3% higher than PD (*p* < 0.0001). Whereas CA and BL values of OC at 100 °C were 25.9% higher than PD (0.34 vs. 0.25 cm; *p* < 0.0001). Therefore, the highest value of springiness was obtained for CA OC-180 and OC-210 when cooking at 100 °C (0.46 cm), and the lowest for PD-30 at 80 °C (0.11 cm).

In terms of cohesiveness, lower values at 100 °C than at 80 °C (*p* = 0.0016) and a value decrease with cooking time (*p* = 0.0157) were observed. Specifically, OC-240 at 100 °C cohesiveness showed the lowest value (0.12; *p* = 0.0163).

Considering PC, no differences were detected in springiness related to the cooking time between 20 and 30 min, but values at 15 min were lower than others (0.36 vs. 0.29 cm; *p* < 0.0001). In addition, PD springiness was lower than BL and CA (0.28 vs. 0.36 cm, respectively; *p* < 0.0001). With regard to cohesiveness, a higher value of PD was detected, but only when cooking for 15 and 20 min (15.8% and 17.6% difference, respectively; *p* < 0.05). In addition, the cohesiveness of chickpeas cooked for 30 min showed a 70.7% lower value than those cooked for 15 or 20 min (0.16 vs. 0.05, respectively; *p* < 0.05; Table 1).

The differences found in the effect of heat treatment on the textural characteristics of the different chickpea varieties used in this study can be attributed to their different structural, morphological, and compositional characteristics. The PD chickpeas are categorized as *desi* (small, wrinkled, and dark-colored) [35,36], whereas BL and CA are *kabuli* varieties (large, smooth-coated, and white to cream-colored). PD chickpeas, due to their smaller diameter, reached the temperature of the cooking medium more quickly than larger chickpeas, thus affecting their structure and textural attributes, such as adhesiveness (greater in PD). However, PD chickpeas presented greater hardness in OC and required longer cooking times for softening, which is attributed to the characteristics of their seed coat and cotyledon structure, where the starch aggregates are embedded in the protein structure [37].

As is well known, the seed coat can prevent seed swelling during cooking; it is a physical obstacle to hydration capacity [38,39]. Wood et al. [28] reported that *kabuli* seeds had a thinner seed coat associated with the thinner palisade and parenchyma layers, which contained fewer pectic polysaccharides and less protein. The outer palisade layer varied in thickness from one to two cells, leading to a textured and sometimes wrinkled appearance of the seed surface. In contrast, the *desi* palisade layers were rigid and extensively thickened.

It is worth mentioning that Kaur et al. [40] and Singh et al. [41] studied the dependence on genotype and cultivar conditions of the physicochemical and textural properties of soaked and cooked chickpeas. They concluded that the lower the seed weight and/or volume, the more compact the structure (seed coat and cotyledon) and the lower the hydration ability. Several authors [40,41] have reported that, together, the swelling capacity and the swelling index are directly correlated to hardness and cohesiveness and indirectly to springiness. An inverse relationship was observed between hydration and cohesiveness. This agrees with our results and could explain the lower elasticity and higher hardness of PD vs. CA and BL.

In addition to the differences attributable to morphology, especially of the seed coat and the cotyledon, a strong relationship between changes in TPA variables and the chickpea biochemical composition has been described. A dependence of rheological parameters on hydration amount, protein and starch contents, and amylose/amylopectin ratio has been previously described by Gómez-Favela et al. [42] and Costa et al. [43]. However, disparity among studies has been also found. Kalefetoğlu et al. [44] described that *kabuli* varieties are characterized by a higher protein amount than *desi* varieties. Whereas Ipekesen et al. [45] and Sahu et al. [46] found no difference in protein content, only in selected amino acids, when comparing *desi* and *kabuli* varieties. Remarkably, Xiao et al. [36] described a higher starch amount in *kabuli* than in *desi*, but, again, no difference in protein concentration.

According to the existing literature, the analysis of the rheological behavior of different chickpea varieties seems to be a complex problem involving multiple factors. Koskosidis et al. [47] and Ozaktan et al. [48] described a strong dependence of chickpea composition with the genotype and sowing, and with climate and soil composition, respectively. Therefore, food processors should be aware of the different components of various chickpea species in order to select specific species for better preparation of pulse-based food products [36].

The cooked chickpeas were evaluated in a sensory analysis to establish the most suitable cooking conditions for the development of the most satisfactory sensory attributes without affecting the integrity of the legume. Wood [28] carried out a literature review on the evaluation of cooking time in pulses, observing a lack of a standard method. The major direct measures used to evaluate cooking time include sensory analysis, tactile methods, spread area ratio, use of the Mattson bean cooker, and white core and glass slide methods.

In the sensory analysis, by a rank order test and descriptive observation, the panelists evaluated the level of satisfaction, taking into account the saltiness and aroma of the chickpeas and considering the integrity of chickpeas in terms of preserved skin. Concerning the texture characteristics, hardness values were used as limiting factors, together with low cohesiveness (sample disintegration while cooking) but not adhesiveness, which has been described as a less limiting factor for the acceptance of the products by consumers [27]. Overall, the softness in the mouth and the soft inner mass of the chickpeas were mainly considered.

In the first sensory analyses, cooked chickpeas that presented a hardness higher than 1.5 N were considered unsuitable for consumption, due to their high chewing resistance. OC chickpeas cooked at 80 °C showed hardness values higher than 3 N, even when cooking was prolonged up to 360 min (data not shown), resulting in a product that was far from “buttery” and flavorful; this may be associated with an incomplete starch gelatinization in cotyledons. Klamczynska et al. [49] reported that the hardness of seeds decreased continuously with increased cooking time. These authors found that all legume starch was fully gelatinized after heating at the boiling point for 70 min. Retrogradation of starch in the legume seeds occurs relatively quickly during cooking and is promoted by extended cooking.

Table 2 shows the results of the sensory analysis corresponding to cooking conditions (discarding OC chickpeas cooked at 80 °C).

The highest score was assigned, independently of the chickpea variety, to OC-180 at 100 °C (*p* < 0.05). With regard to PC, the highest score was given to 20 min, also independently of the chickpea variety (*p* < 0.05, Table 2). Interestingly, no panelist issued a negative judgment related to the texture or taste. In addition, in the case of PC chickpeas cooked for longer than 30 min, skin and/or integrity loss was observed in a high percentage; therefore, they were also eliminated from further study. In addition, PC-30 and PC-60 chickpeas showed the lowest cohesiveness values.

Besides cooking, other factors, such as growing conditions and variety/cultivar, can affect sensory parameters [27]. Cobos et al. [50], together with Chigwedere et al. [51], observed that the genotype effect was the greatest source of variability in all grain quality studies; hence, a high genetic gain of grain quality is expected for nutritional, physical, and sensory traits in chickpea. In addition, no clear relationship was found between the sensory and physicochemical properties. The authors concluded that buttery texture, graininess, and hardness were the specific variables adequate to evaluate sensory quality in chickpeas to minimize the required time for panel testing.

The objective of this part of the study is to select cooked chickpeas that are intact, have sensory characteristics suitable for consumption, and could be subjected to freeze-drying for their preservation and subsequent use in various preparations after rehydration. Therefore, considering the sensory evaluation results, it was decided to continue the study with the three varieties of chickpeas, but only with the treatments: OC-180 at 100 °C and PC-20, whose TPA characteristics were as follows: hardness: ≤0.18 ± 0.010 N, springiness: ≥0.30 ± 0.010 cm, and cohesiveness: ≤0.15 ± 0.012. Nevertheless, chickpeas cooked under OC-210 conditions also presented satisfactory sensory characteristics, although the bean cohesiveness and integrity tended to decrease with increasing cooking time. This same tendency was also observed in PC chickpeas cooked longer than 20 min.

### 3.2. Rehydration Kinetics of Cooked Freeze-Dried Chickpeas

**Figure 3:**
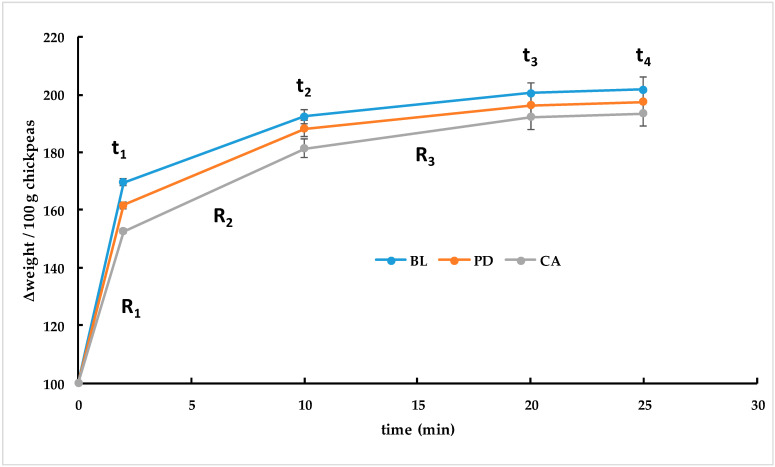
Rehydration curve model of different varieties of cooked and freeze-dried chickpeas. Chickpea varieties from Spain: PD = *Pedrosillano*; BL = *Blanco Lechoso*; CA = *Castellano*.

The freeze-dried chickpeas were kept vacuum packed at −20 °C to avoid changes in the remaining moisture content and in the oxidative stability. The rehydration process, including weight gain and its time dependence, was non-linear. Independently of the chickpea variety, selected cooking conditions (OC-180 at 100 °C and PC-20), and the rehydration conditions (distilled water or 2% NaCl solution at 20–22 °C or 90–100 °C), three stages of rehydration were seen (Figure 3). Firstly, a sudden weight gain can be observed (R_1_; t_1_ = 0–2 min), related to a quick inclusion of water by the external structure of the chickpea (the highest sorption velocity corresponding to the highest slope value). Secondly, a medium velocity of rehydration was seen, which could be related to a subsequent inclusion of water within the internal structure of the chickpea, with the degree of incorporation of external water lower than R_1_ but still considerable (R_2_; t_2_ = 2–10 min). Thirdly, a low weight gain period was observed, which could be related to a structural reorganization to a higher extent and to water inclusion as a secondary process (R_3_; t_3_ = 10–20 min). The third period was followed by an equilibrium (t_4_ > 20 min), in which no significant changes in weight gain were detected. Consequently, the maximum weight gain at the end of rehydration can be estimated at 20 min. Similar curves of rehydration have been previously observed by several authors in different vegetables and pulses [42,52]. It has been reported that the rehydration is associated with hysteresis due to cellular and structural disruption [52].

When only considering the chickpea variety, it was observed that the percentage weight gain of the BL variety was 7.77% and 6.05% higher than CA and PD at R_1_ (*p* < 0.05) and 4.89% and 2.86% at R_2_ (*p* = 0.0713), respectively. At the end of the rehydration process (R3), CA chickpeas showed slightly lower rehydration values (% weight gain) than the other chickpea varieties (Figure 3).

Water absorption kinetics have been widely studied to evaluate the changes in the microstructure and physicochemical properties of food matrices. In fact, specifically for chickpeas, the rehydration process has been previously described by several authors who established Peleg’s [42,53], Weibull’s [54,55], or Fick’s [56] models as the most accurate for rehydration process prediction. Nevertheless, to compare results, it is necessary to consider the differences between experimental models. For legumes, most published studies analyze the rehydration of seeds during soaking or the cooking process [42], whereas the present work deals with the rehydration of previously cooked and then freeze-dried chickpeas.

**Figure 4:**
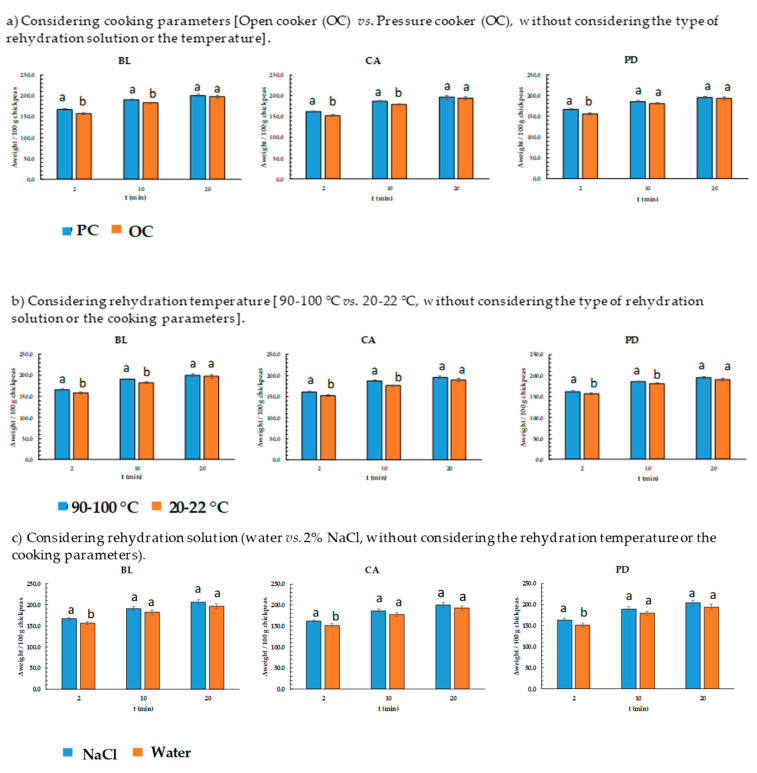
Weight gain of freeze-dried chickpeas considering cooking conditions, rehydration temperature, and solution. Chickpea varieties from Spain: PD = *Pedrosillano*; BL = *Blanco Lechoso*; CA = *Castellano*. Values without a common letter within the same rehydration time differ significantly (*p* < 0.05).

Figure 4 compares, for each chickpea variety, the mean value of weight gain at the end of stage R1 (2 min), R2 (10 min), and R3 (20 min), considering the selected cooking conditions (OC-180 at 100 °C vs. PC-20, Figure 4a), the temperature (20–22 °C vs. 90–100 °C, Figure 4b), and the rehydration medium composition (distilled water vs. 2% (*w/v*) NaCl solution, Figure 4c). In all chickpea varieties, PC rehydrated at a faster rate at the R1 stage than OC, although the differences in weight gain of each variety at the end of R3 were not statistically significant (Figure 4a).

Independently of the chickpea variety and the cooking procedure, considering R_1_, higher values of weight gain were observed when the rehydration was carried out with 2% NaCl (16.6% difference, Figure 4c) solution and/or with a heated medium (11.0% difference; *p* < 0.05, Figure 4b). With regard to R_2_, higher values of weight gain were obtained when using a heated (90–100 °C) rehydration media as compared to a room-temperature (20–22 °C) one (12.4% difference; *p* < 0.05), and a statistical tendency was detected regarding rehydrating solution (14.4% difference; *p* = 0.088) (Figure 4c).

The differences among chickpea varieties could be related to differences in seed morphology and physico-chemical characteristics such as the size and shape of the seed, rate of starch gelatinization, the ratio of the soluble/insoluble pectates, lignification of middle lamella, seed coat characteristics, and lipid structure, among others [42]. In addition, the nature and amounts of non-starch constituents, which act as a physical barrier to the swelling of starch granules, may influence the rate of water uptake during the cooking and, perhaps, also during rehydration after a freeze-drying process. Additionally, genotype and cultivar conditions have been described to affect rehydration parameters [42], which is in line with our results from different chickpea varieties from different locations.

The differences between the rehydration rate of chickpeas treated in OC and PC can be attributed to the higher intensity and homogeneity of the cooking treatments performed at high pressure [33]. Thus, the rate of hydration, hydrogen bond disruption, and other phenomena associated with the cooking process of the seeds will be enhanced in PC and, consequently, in subsequent rehydration after freeze-drying of the cooked product. In addition, the thickness of the palisade layer and the lignin and cellulose content of the seed coat and the cotyledon cell walls, relevant structures involved in the cooking quality, will be affected in a higher proportion in PC. A more intense heat treatment should imply a higher degree of hydrolysis (or other breaking-down processes of macromolecules). The more the macromolecules are degraded, the easier the water inclusion (diffusion) should be. An equilibrium exists between water inclusion (depending on membrane/skin permeability, starch structure, and seed size) and loss of components [43,56].

Regarding the impact of the temperature of the rehydration medium, our results agree with Krokida and Marinos-Kouris [57]. These authors established that, in general, the water temperature positively influenced the rehydration rates and the equilibrium moisture content of dehydrated products. In this line, Monteiro et al. [58] described a linear relationship between water temperature and weight gain of chickpeas during rehydration.

Concerning the increased rehydration rate of chickpeas rehydrated with 2% NaCl, it has been reported that sodium salts increase water absorption, leach solids and result in softness in the cooked beans, and increase pectin solubilization, decreasing Ca and Mg (ions that bind pectin), when compared to distilled water or the other salts [59].

From the data obtained in the present work, it can be deduced that, although differences were observed in the water sorption rate at the beginning of rehydration, in general, all seeds showed a high degree of water uptake at the end of the rehydration process. This fact could be due to the high porosity, low density, and spongy structure induced by the freeze-dying process, facilitating rehydration. On the other hand, the freeze-drying process induces limited changes in the cell network, which would also facilitate rehydration [60]. Therefore, it is considered that, although there are differences in seed structure derived from factors specific to chickpea variety and the applied thermal process, the freeze-drying process would result in suitable matrices for water uptake during rehydration. Aravindakshan et al. [60] reported that the type of drying process together with the temperature of rehydration, the nature of pores, and the state of water-imbibing constituents influenced the rehydration characteristics of dried beans. Liu et al. [8] reported that freeze-drying was the best drying procedure as compared to others.

The results of the sensory analysis are shown in Table 3. In terms of rehydration weight gain, rehydration at 0–100 °C and with NaCl solution, independently of the cooking conditions (OC and PC), were given higher scores than room temperature and only-water rehydration protocols (*p* < 0.01), as samples had a softer texture and a more intense flavor. However, all rehydrated chickpeas were described as having the characteristics of cooked legumes. These results can be related to the findings of Ulloa et al. [61], who reported that the rate of absorbed water determines the sensory properties.

As is well known, the basic objective of drying food products is the elimination of water from solids to a level at which microbial spoilage is minimized. In this line, freeze-drying has considerable advantages. Chen et al. [62] observed that freeze-drying-based processing technologies are especially suitable for producing products containing abundant nutrients and functional ingredients due to a drying environment with lower temperature and oxygen content. In fact, several studies regarding the development of dehydrated chickpea snacks obtained by sequential hydration, cooking, and drying processes have been found in the literature [58,63]. Ulloa et al. [61] established that instant whole beans obtained by drying could represent a good-quality bean-based product for new market opportunities in the functional food and nutraceutical industry. The authors described that the rehydration characteristics of a dehydrated product reflect the physical and chemical changes that occurred during dehydration, thus being suitable to be used as a quality index. These changes are influenced by the composition, the conditions during drying, and any pre-treatment to which the products have been subjected. Remarkably, the authors also established that the amount and rate of absorbed water determine the sensorial properties and the preparation time required by the consumer.

Therefore, more research is required to determine alternative ways to incorporate chickpea-based ingredients into products and understand their behavior when used in different food categories (beverages, snacks, dairy-like products, etc.). In the present study, after testing the acceptance of cooked freeze-dried, and rehydrated chickpeas, their use is proposed in the preparation of a quick cooking dish based on a traditional culinary preparation in Spain, *Cocido Madrileño*. However, this use is only intended as an example of how complex traditional dishes with several ingredients could be prepared from freeze-dried cooked chickpeas.

### 3.3. Analysis of the Cooking and Rehydration Parameters and Sensory Characteristics of the Other Components of the Cocido Dish

Table 4 shows the results of the sensory analysis of other ingredients of a traditional *Cocido Madrileño* dish [16,17] (different pieces of beef and chicken meat, dry-cured ham and pork belly, and vegetables) after cooking (OC 100 °C and PC, at different times), freeze-drying, and rehydration (2% NaCl solution) (Figure 2).

Juicy chicken meat with a good appearance and flavor was obtained with OC-180; however, OC-60 led to poor development of aroma, taste, and appearance, and the meat texture was defined as tough. Similar to chickpeas, PC cooking shortened the time required for the development of adequate organoleptic characteristics. Samples cooked for 30 min presented a tough texture, poor development of flavor and aroma, and significantly (*p* < 0.05) lower sensory evaluation values than samples cooked for 90 min, which presented a soft but dry and disintegrated texture, with a lack of cohesion and less pleasant flavor and aroma. In all cases, the cooked freeze-dried samples presented sensory characteristics after hydration very similar to those shown when freshly cooked. In all cases, a greater development of flavor and aroma was shown with longer cooking times. Additionally, after 60 min in PC and 90 min in OC, rehydrated chicken meat showed satisfactory sensory qualities.

Overall, in agreement with our results, related to cooking conditions, it has been described that the higher the temperature and the lower the moisture, the more heterocyclic flavor compounds that will be formed. In addition, PC chicken meat has been described to be closer to roasted or grilled chicken meat than boiled [64]. It should be noted that our results agree with the higher temperature and time, but that, in the case of PC, although the literature has reported better flavor compounds, the panelists described the chicken meat as less succulent both before and after freeze-drying.

Regarding beef flank and shank sensory analyses, shorter cooking times (OC-60 and PC-30) led to tough and barely chewable meat. Whereas meat cooked for longer times (OC-180 and PC-90) was soft and juicy and had greater flavor development. Unlike PC-90 meat, the panelists described an intense sapid and aromatic development of meat flavor for OC-180 products. However, in the descriptive analysis of PC-90, a tender, juicy, and smooth texture was described. OC-60 and OC-90 led to poor development of aroma, taste, and appearance, and the meat texture was defined as tough and barely chewable. The differences in flavor intensity and cooking times could be due to the progress of the primary lipid degradation and Maillard reaction products, associated with beef flavor [65]. The results obtained are in line with Juárez et al. [66] regarding the importance of endpoint temperature in beef cookery, which greatly impacts consumer satisfaction. Again, the cooked freeze-dried samples presented sensory characteristics after hydration that were very similar to those shown when freshly cooked. In agreement with the literature, the obtained results strengthen the consideration of freeze-drying as a high-quality preservation method that maintains the organoleptic characteristics of meat and meat products [60,67,68].

Regarding the meat texture, in both OC and PC, deficient collagen hydrolysis could explain the described behavior, according to Etheritong and Sims [69] and Sims and Bailey [70].

When meat is cooked at a slow rate of heating, tenderness and juiciness are improved, although cooking losses increase. Juiciness is affected by exudation and diffusion in moist heat cookery (e.g., stewing or braising). In general, the muscle structural composition determines the cooking effect on tenderness. In the case of muscles with high connective tissue content, such as the meat pieces used in this study, this meat profits from moist cooking due to collagen gelatinization [66].

Although the purpose of incorporating a portion of bone-in cured ham in the traditional dish [16,17] is less about consumption than about contributing to the sapid and aromatic development of the cooking broth, the cured ham cooked at longer times (≥90 min in OC and 60 min in PC) was more palatable and softer than that cooked at shorter times, and both the appearance and the integrity of this product were maintained when rehydrated after freeze-drying. Similar results were obtained for pork belly. A piece (about 60 g/L) of this meat portion is also added in the traditional *Cocido Madrileño* [16,17], both to add flavor and to be consumed if desired. It is worth noting the good rehydration of this portion, despite its fat content when rehydrated with 2% NaCl solution (90–100 °C) after freeze-drying.

To increase the broth flavor [16,17], cow leg-bone (Figure 2) was included. Such a portion was added for all methods during the described cooking times but was not considered in sensory analysis nor freeze-dried.

Potato, carrot, leek, and turnip showed very similar behavior. Although the size of the pieces should be considered (in our case, potato and turnip less than 70 mm and carrot 2–3 cm in diameter), OC-60 and PC-30 vegetables showed harder texture and lower sapid and aromatic development but a more compact appearance and a lower sensory score than OC-180 and PC-90 (*p* < 0.05). In additional tests carried out with longer cooking times (200 min), a disintegration of the matrix structure was detected, especially in potatoes and carrots. Again, the cooked freeze-dried samples presented sensory characteristics after hydration very similar to those shown when freshly cooked (Table 4). These results are in line with the findings of Liu et al. [8] on the suitability of freeze-drying in maintaining the optimal sensory and nutritional properties of rehydrated vegetables. In addition, the results are in synchrony with the findings of several authors who studied the effect of heat treatment on vegetables such as potatoes. Changes in the potato tuber microstructure and texture during cooking have been mainly associated with the gelatinization behavior of starch through the cell wall, but the middle lamella structural components also play a role [71,72]. A study by Paulus and Saguy [73] on the effect of heat treatment on the quality of cooked carrots suggested that boiling for 70 min led to the best sensory evaluation. In addition, other authors have attributed the softening to cellular dehydration and separation in the tissue [74] as well as the membrane disruption associated with the loss of turgor [75].

Parallel to the aromatic and sapid development of the meat and vegetable portions, the cooking water (broth) presented a flavor with higher intensity, closer to that of the broth of the traditional product, at longer cooking times (Table 4), both in OC and PC cooking. It should be noted that, in the PC-90 broth, although qualified with the highest scores in the ordination analysis, the detection of a slight “overheated” aftertaste was mentioned in the descriptive analysis. Such an aftertaste was not described for OC-180. In all cases, the freeze-dried broths after rehydration presented sensory characteristics very similar to those described in the freshly cooked broth and, in the case of the broths obtained at longer times (OC-180 and PC-90), sapid matrices very close to those of traditional broth were described.

Broth-related results were in synchrony with those obtained in previous works. Cambero et al. [23,24] and Wang et al. [76] observed that the sensory quality of beef broths increased with the increase in the concentration of nitrogen in peptides (molecular weight > 600 Da), small non-amino acid nitrogen compounds (<600 Da), creatine, and inosine, adenosine and guanosine 5’-monophosphate, and substances from the earlier steps of Maillard reactions, thus being involved in the obtaining of suitable beef broth flavor. In fact, the development of off-flavors, especially warmed-over flavors, was related to an intensification of the cooking treatment. Cambero et al. [23,24] and Pereira-Lima et al. [77] for beef, and Pérez-Palacios et al. [78] and Zhang et al. [79] for chicken, described an implication of over-cooking, with bitter amino acids and peptides, among others.

In line with tradition, cabbage, *chorizo,* and *morcilla* were cooked separately (Figure 2) so as to not interfere with the broth taste [16,17]. OC-30 and PC-15 cabbage showed the best sapid characteristics (Table 4). Shorter cooking times resulted in texture toughening, and longer cooking times, especially PC, led to good flavors but, to a higher or lower extent, the disintegration of the product. As with other ingredients, freeze-drying did not produce a significant effect on the sensory characteristics of cabbage cooked at the established times, and after rehydration, sensory attributes similar to those of the freshly cooked product were described (Table 4).

Regarding cabbage, it has been described that mild cooking minimizes tissue damage and maximizes beneficial isothiocyanate formation and the retention of the active myrosinase enzyme, whose activity on the cabbage glucosinolates produces several compounds with chemo-protective effects [80,81]. On the other hand, it has also been reported that freeze-drying is the method in which the contents of chlorophyll and saponins are kept at a high level in vegetables [8].

In the case of *chorizo* and *morcilla*, to maintain their integrity, only OC cooking was considered. The highest rating was achieved when both products were cooked for 15 min. When longer cooking times were considered, dry texture and rancidity aftertaste, which became more evident after the product was freeze-dried and rehydrated, were detected. It must be considered that they are processed ground meat products with a high content of fat and, consequently, very susceptible to oxidation. Accordingly, Toldrá and Reig [82] and Lorenzo et al. [83] described a dependence on the heating extent, moisture content, proteolysis degree, and the content of fat and connective tissue on the sensory attributes of pork sausages. A higher surface of the components in contact with the atmospheric oxygen and light must be expected; therefore, oxidative processes could develop more rapidly. Thus, the freeze-drying and the rehydration optimization of *chorizo* and *morcilla* require further study.

From the results obtained from the sensory analyses to determine the most convenient conditions for the development of satisfactory attributes (Table 4), it is deduced that the cooking process of the different ingredients of *Cocido Madrileño* could be unified so that the vegetable ingredients, bone-in cured ham, chicken, and beef would be OC-180 cooked, obtaining a broth with sensory attributes similar to the traditional dish. An equally valid alternative would be cooking in PC-90. These ingredients maintain their sensory quality by rehydrating after freeze-drying. To these ingredients, chickpeas would be added (preferably of the BL variety, although other studied varieties could also be used) and freeze-dried after OC-180 or PC-20 cooking. Additional ingredients, cooked separately, require shorter times, such as cabbage (OC-30 or PC-15) and pork sausage (OC-30).

### 3.4. Preparation of a Freeze-Dried Cooked Cocido as an Easy-to-Prepare Dish: Dish Assembly and Packaging Proposal

Once the best cooking conditions for each ingredient, considering the development of appropriate sensory attributes, were selected (Table 2, Table 3 and Table 4), experiments were carried out to prepare a complete *Cocido Madrileño* dish from cooked and freeze-dried ingredients with the objective of obtaining an easy-to-prepare dish.

As mentioned, *Cocido Madrileño* is a traditional meal, currently restricted to dishes in which chickpeas play the leading role and different vegetables and meats, although essential, enhance the dish and confer the specific flavor [16]. This proposal aims to analyze the potential of formulating complex dishes from cooked and then freeze-dried ingredients of a different nature to offer healthy dishes that require minimal preparation.

In Spain, when the word “*Cocido*” is heard, memories and emotions are evoked. Historically, the root of the *Cocido* dish was assessed to be a plate cooked by Jewish families each Friday, to be eaten the next day, on the *Sabbath*, the sacred day of the Jewish week. It was named *Adafina* and included lamb meat, chickpeas, and other vegetables. In 711, the Arabs revolutionized the *Cocido* with their more sophisticated agriculture: softer and pulpier chickpeas and several vegetables were used in their *Cocido*, named “*Tajine*” [17]. Currently, the name has been restricted to dishes in which chickpeas play the leading role and different vegetables and meats, although essential, enhance the dish and confer the specific flavor [16]. Strictly, the consumption protocol of the *Cocido* establishes that the first course must be a soup of thin noodles, followed by the degustation of the chickpeas, as the second course. Meat and other vegetables constitute the third course [17]. However, nowadays, chickpeas, vegetables, and meat are served together as a second course, after the soup, which was considered in the assembly of the freeze-dried dish under study.

**Figure 5:**
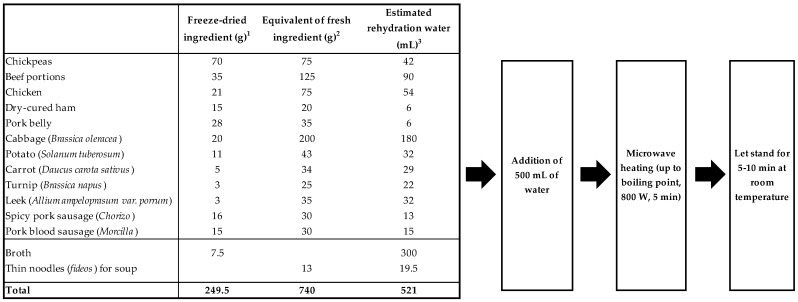
Proposal for the assembly of a *Cocido* dish from cooked and freeze-dried components and the rehydration procedure to prepare a reconstituted tasty meal. ^1^ Freeze-dried products after cooking in an open cooker for selected times. ^2^ The equivalent weight of the fresh product before culinary treatment (in the case of chickpeas, this refers to the weight of the dry legume). ^3^ Estimation of the rehydration water required for each product. In the case of chickpeas, the rehydration water for cooking was considered. For the replacement of the broth, the dry extract of the cooking water of the ingredients was considered (2.5%).

In these experiments, an attempt was made to establish the processing conditions that could be replicated in the industrial processing of this dish. In this case, in contrast to traditional culinary processing, and taking into account the more appropriate processing conditions established, the chickpeas were cooked separately for greater control of the heating time and temperature. Meat portions and vegetables (except cabbage, *morcilla,* and *chorizo,* which were cooked separately) were cooked (with 2% salt in an open cooker) together to obtain a suitable broth (Table 4). Each ingredient was freeze-dried individually. Trying to closely imitate the customary protocol at a laboratory level, for rehydration of the ingredients, the freeze-dried broth and the quick-to-cook thin noodles were placed at the bottom of a beaker and, over them, a perforated basket (hole diameter lower than chickpea diameter, 3 mm^2^) containing chickpeas, vegetables, and meat. A total of 500 mL of water was added and heated in a microwave oven (800 W) for 5 min. As shown in Figure 5, the added water was calculated by considering both the rehydration water of each component and the obtainment of a bowl of soup (about 300 mL).

After 5 min of heating, the beaker was removed from the microwave and left to stand for 10 min. The basket with the ingredients was removed, and the noodles and broth (up to the consumer’s preference) were placed in a dish. Then, the basket was placed back in the beaker with the remaining broth. Thus, the rest of the ingredients (chickpeas, vegetables, and meat) were kept juicy until they were eaten as a second course.

Sensory analysis was immediately carried out through a descriptive analysis with panelists who were familiar with the traditional dish. The panelists were asked for an evaluation of the whole reconstituted dish compared to an original *Cocido Madrileño* (firstly the taste and aroma of the soup, and secondly the taste, aroma, and texture of the other ingredients) and punctuation of each ingredient individually. In all cases, the rehydrated products were evaluated with sensory characteristics similar to those of the traditional dish, although for *chorizo* and *morcilla*, the panelists described a slightly rancid taste and a lightly dry and disintegrated texture.

The fast food that can be found on the market is not always as healthy as desirable. If a freeze-dried complex dish (noodle soup, vegetables, and meat) with easy rehydration could be achieved, it would offer the consumer a novel product (there is no similar product on the market) that is easy to preserve (it can be stored at room temperature), requires minimal preparation (limited to the addition of water and short heating in a microwave), has reputed sensory quality and richness of ingredients (legumes, vegetables, and meat products), and is nutritionally well valued, in coherence with the Mediterranean diet [84].

The authors are aware that further studies related to the shelf-life and stability of the product when stored in a container are mandatory. Nevertheless, it must be remembered that one of the objectives of this work was to offer plausible alternatives of an easy-to-prepare reconstituted *Cocido* dish that could be widely produced. We are working on the design of a container that facilitates rehydration, heating, and, above all, the consumption of the ingredients in the traditional way: in two or three separate courses.

## 4. Conclusions

It is possible to elaborate *easy-to-prepare* dishes containing chickpeas and various ingredients (vegetables and different meat products) prepared with products cooked at their optimum point and then freeze-dried. These dishes can be a commercial alternative to “fast food”, since they are easily rehydrated and ready for consumption in a few minutes, providing a varied and complete supply of nutrients. As proof, in the case of the preparation of the traditional Spanish dish “*Cocido Madrileño*”, a product with a sensory quality similar to the traditional dish ready requiring about 15 min of preparation was obtained.

However, it is necessary to carry out more research to study the stability and shelf-life of these products, as well as to determine the economic viability.

## Figures and Tables

**Table 1:** Texture profile analysis of chickpeas cooked under different systems and times.

Cooking System	Temperature (°C)	Time (min)	Hardness (N)			Adhesiveness (N×s, ×10^1^)			Springiness (m, ×10^2^)			Cohesiveness		
			BL	CA	PD	BL	CA	PD	BL	CA	PD	BL	CA	PD
**OC**	**80**	**30**	34.8 ± 6.76 ^a,β^	65.6 ± 9.01 ^a,α^	29.8 ± 2.92 ^a,γ^	−0.04 ± 0.027 ^a^	−0.03 ± 0.007 ^a^	−0.02 ± 0.021 ^a^	0.20 ± 0.020 ^b,α^	0.25 ± 0.050 ^c,α^	0.11 ± 0.039 ^c,β^	0.15 ± 0.041 ^ab^	0.11 ± 0.008 ^ab^	0.14 ± 0.021 ^ab^
		**60**	21.6 ± 2.50 ^b,β^	42.1 ± 5.57 ^b,α^	21.9 ± 3.15 ^b,β^	−0.05 ± 0.019 ^a^	−0.03 ± 0.015 ^a^	−0.03 ± 0.023 ^a^	0.21 ± 0.031 ^b,β^	0.28 ± 0.011 ^b,α^	0.13 ± 0.058 ^c,γ^	0.23 ± 0.039 ^a^	0.11 ± 0.009 ^ab^	0.13 ± 0.020 ^ab^
		**90**	19.8 ± 3.52 ^b,α^	21.0 ± 9.00 ^c,α^	13.8 ± 3.48 ^c,β^	−0.09 ± 0.044 ^a^	−0.06 ± 0.018 ^a^	−0.04 ± 0.013 ^a^	0.22 ± 0.039 ^b,β^	0.30 ± 0.043 ^b,α^	0.13 ± 0.039 ^c,γ^	0.17 ± 0.049 ^a^	0.17 ± 0.008 ^a^	0.18 ± 0.011 ^a^
		**120**	12.8 ± 4.31 ^b,β^	17.2 ± 6.30 ^c,α^	13.7 ± 6.43 ^c,β^	−0.04 ± 0.031 ^a^	−0.06 ± 0.017 ^a^	−0.06 ± 0.030 ^a^	0.27 ± 0.051 ^ab,α^	0.33 ± 0.066 ^b,α^	0.21 ± 0.038 ^b,β^	0.18 ± 0.038 ^a^	0.19 ± 0.014 ^a^	0.19 ± 0.031 ^a^
		**150**	10.5 ± 1.88 ^b,β^	19.3 ± 7.14 ^c,α^	8.90 ± 1.79 ^c,β^	−0.03 ± 0.020 ^a^	−0.02 ± 0.011 ^a^	−0.03 ± 0.013 ^a^	0.30 ± 0.033 ^a,β^	0.41 ± 0.019 ^a,α^	0.23 ± 0.078 ^ab,γ^	0.15 ± 0.048 ^ab^	0.15 ± 0.041 ^a^	0.14 ± 0.021 ^ab^
		**180**	6.72 ± 1.52 ^c,β^	18.3 ± 4.01 ^c,α^	11.8 ± 5.33 ^c,β^	−0.09 ± 0.041 ^a^	−0.05 ± 0.019 ^a^	−0.03 ± 0.023 ^a^	0.33 ± 0.074 ^a,α^	0.37 ± 0.033 ^a,α^	0.23 ± 0.030 ^ab,β^	0.19 ± 0.090 ^a^	0.16 ± 0.071 ^a^	0.18 ± 0.009 ^a^
		**210**	5.24 ± 1.22 ^c,β^	8.83 ± 4.01 ^d,β^	15.8 ± 2.98 ^c,α^	−0.04 ± 0.026 ^a^	−0.03 ± 0.016 ^a^	−0.02 ± 0.023 ^a^	0.30 ± 0.064 ^a,α^	0.37 ± 0.071 ^a,α^	0.23 ± 0.041 ^ab,β^	0.24 ± 0.048 ^a^	0.17 ± 0.043 ^a^	0.13 ± 0.041 ^ab^
		**240**	3.64 ± 1.12 ^c,β^	12.2 ± 3.00 ^d,α^	10.2 ± 3.74 ^c,α^	−0.05 ± 0.022 ^a^	−0.03 ± 0.007 ^a^	−0.04 ± 0.011 ^a^	0.25 ± 0.014 ^b,α^	0.35 ± 0.099 ^ab,α^	0.18 ± 0.058 ^b,β^	0.15 ± 0.014 ^ab^	0.20 ± 0.032 ^a^	0.17 ± 0.011 ^a^
	**100**	**30**	14.1 ± 4.70 ^b,α^	3.25 ± 0.51 ^e,β^	9.98 ± 2.92 ^c,α^	−0.16 ± 0.061 ^b,β^	−0.11 ± 0.001 ^b,γ^	−0.60 ± 0.101 ^b,α^	0.23 ± 0.059 ^b,α^	0.25 ± 0.010 ^c,α^	0.17 ± 0.060 ^ab,γ^	0.16 ± 0.021 ^ab^	0.12 ± 0.041 ^ab^	0.17 ± 0.020 a
		**60**	7.45 ± 1.50 ^c,α^	2.08 ± 0.03 ^f,β^	6.49 ± 0.15 ^d,α^	−0.19 ± 0.091 ^b,β^	−0.12 ± 0.003 ^b,γ^	−1.07 ± 0.121 ^c,α^	0.27 ± 0.058 ^ab,β^	0.32 ± 0.004 ^b,β^	0.23 ± 0.059 ^ab,β^	0.15 ± 0.011 ^ab^	0.14 ± 0.033 ^ab^	0.17 ± 0.019 ^a^
		**90**	1.42 ± 0.700 ^d,α^	1.02 ± 0.011 ^g,β^	1.14 ± 0.137 ^e,α^	−0.20 ± 0.081 ^bc,β^	−0.18 ± 0.081 ^c,β^	−1.90 ± 0.301 ^d,α^	0.33 ± 0.060 ^a,α^	0.35 ± 0.061 ^ab,α^	0.23 ± 0.049 ^ab,β^	0.17 ± 0.023 ^ab^	0.15 ± 0.021 ^ab^	0.17 ± 0.031 ^a^
		**120**	0.39 ± 0.018 ^e^	0.28 ± 0.013 ^h^	0.27 ± 0.010 ^f^	−0.20 ± 0.071 ^bc,β^	−0.20 ± 0.023 ^c,β^	−1.60 ± 0.173 ^d,α^	0.35 ± 0.060 ^a,α^	0.37 ± 0.059 ^a,α^	0.27 ± 0.058 ^a,β^	0.15 ± 0.011 ^ab^	0.17 ± 0.041 ^ab^	0.16 ± 0.021 ^a^
		**150**	0.35 ± 0.019 ^e^	0.26 ± 0.140 ^h^	0.20 ± 0.014 ^f^	−0.21 ± 0.055 ^bc,β^	−0.21 ± 0.004 ^c,β^	−1.57 ± 0.151 ^d,α^	0.37 ± 0.059 ^a,α^	0.40 ± 0.080 ^a,α^	0.27 ± 0.059 ^a,β^	0.17 ± 0.022 ^ab^	0.14 ± 0.023 ^ab^	0.14 ± 0.019 ^ab^
		**180**	0.19 ± 0.013 ^e^	0.20 ± 0.010 ^h^	0.18 ± 0.010 ^f^	−0.21 ± 0.039 ^bc,β^	−0.19 ± 0.090 ^c,β^	−1.43 ± 0.423 ^d,α^	0.37 ± 0.039 ^a,αβ^	0.45 ± 0.030 ^a,α^	0.33 ± 0.021 ^a,β^	0.15 ± 0.014 ^ab^	0.16 ± 0.018 ^ab^	0.16 ± 0.009 ^ab^
		**210**	0.19 ± 0.020 ^e^	0.18 ± 0.011 ^h^	0.16 ± 0.015 ^f^	−0.29 ± 0.061 ^c,β^	−0.22 ± 0.013 ^bc,β^	−1.60 ± 0.464 ^d,α^	0.37 ± 0.044 ^a,β^	0.47 ± 0.029 ^a,α^	0.30 ± 0.018 ^a,γ^	0.14 ± 0.013 ^b^	0.15 ± 0.019 ^ab^	0.14 ± 0.019 ^ab^
		**240**	0.15 ± 0.017 ^e^	0.16 ± 0.015 ^h^	0.11 ± 0.031 ^f^	−0.25 ± 0.071 ^c,β^	−0.26 ± 0.043 ^c,β^	−1.24 ± 0.321 ^bc,α^	0.23 ± 0.014 ^b,β^	0.35 ± 0.027 ^ab,α^	0.23 ± 0.013 ^ab,β^	0.11 ± 0.011 ^b^	0.12 ± 0.009 ^b^	0.12 ± 0.011 ^b^
**PC**	**120**	**15**	0.19 ± 0.011	0.18 ± 0.020	0.19 ± 0.019	−0.17 ± 0.018 ^a^	−0.16 ± 0.013 ^a^	−0.14 ± 0.018 ^a^	0.33 ± 0.039 ^b,α^	0.30 ± 0.031 ^b,α^	0.23 ± 0.022 ^b,β^	0.16 ± 0.012 ^a,β^	0.16 ± 0.010 ^a,β^	0.19 ± 0.020 ^a,α^
		**20**	0.18 ± 0.013	0.18 ± 0.010	0.17 ± 0.010	−0.22 ± 0.010 ^b,β^	−0.17 ± 0.010 ^a,α^	−0.26 ± 0.013 ^b,β^	0.37 ± 0.045 ^ab,α^	0.37 ± 0.060 ^ab,α^	0.30 ± 0.013 ^a,β^	0.14 ± 0.020 ^a,β^	0.14 ± 0.011 ^a,β^	0.17 ± 0.010 ^a,α^
		**30**	0.17 ± 0.010	0.17 ± 0.011	0.16 ± 0.070	−0.30 ± 0.020 ^c,β^	−0.27 ± 0.017 ^b,α^	−0.44 ± 0.011 ^c,γ^	0.40 ± 0.011 ^a,α^	0.39 ± 0.020 ^a,α^	0.31 ± 0.010 ^a,β^	0.05 ± 0.010 ^b^	0.04 ± 0.010 ^b^	0.05 ± 0.009 ^b^

OC = open cooker; PC = pressure cooker. Chickpea varieties from Spain: PD = *Pedrosillano*; BL = *Blanco Lechoso*; CA = *Castellano*. a,b… Different letters within the same column for the same cooking system differ significantly (*p* < 0.05). α,β… Different letters within the same row for the same texture parameter differ significantly (*p* < 0.05).

**Table 2:** Sensory analysis of chickpeas cooked under different systems and times.

Cooking Parameters ^1^		Sum of Ranks ^2^					
		Chickpea Variety ^3^					
System	t (Min)	PD		BL		CA	
**OC**	**90**	10	^c^	11	^c^	10	^c^
	**120**	17	^bc^	17	^bc^	18	^bc^
	**150**	35	^ab^	27	^ab^	26	^ab^
	**180**	49	^a^	53	^a^	51	^a^
	**210**	45	^a^	39	^ab^	43	^ab^
	**240**	33	^ab^	42	^ab^	41	^ab^
**PC**	**15**	27	^ab^	28	^ab^	28	^ab^
	**20**	36	^a^	35	^a^	34	^a^
	**30**	18	^bc^	17	^bc^	19	^bc^
	**60**	9	^c^	10	^c^	9	^c^

^1^ OC = open cooker; PC = pressure cooker. ^2^ Sum of ranks; values without a common letter within the same column for the same cooking system differ significantly (*p* < 0.05). ^3^ Chickpea varieties from Spain: PD = *Pedrosillano*; BL = *Blanco Lechoso*; CA = *Castellano*.

**Table 3:** Sensory analysis of chickpeas cooked under selected conditions, freeze-dried, and rehydrated under different conditions.

Cooking Parameters ^1^		Rehydration Parameters		Sum of Ranks ^2^					
				Chickpea Variety ^3^					
System	t (Min)	% NaCl	T (°C)	PD		BL		CA	
**OC**	**180**	**0**	**20–22**	9	^c^	10	^c^	10	^c^
		**2**	**20–22**	19	^bc^	19	^bc^	17	^bc^
		**0**	**90–100**	26	^ab^	26	^ab^	28	^ab^
		**2**	**90–100**	36	^a^	35	^a^	35	^a^
**PC**	**20**	**0**	**20–22**	11	^c^	10	^c^	12	^c^
		**2**	**20–22**	16	^bc^	18	^bc^	18	^bc^
		**0**	**90–100**	29	^ab^	26	^ab^	25	^ab^
		**2**	**90–100**	34	^a^	36	^a^	35	^a^

^1^ OC = open cooker; PC = pressure cooker. ^2^ Sum of ranks; values without a common letter within the same column for the same cooking system differ significantly (*p* < 0.05). ^3^ Chickpea varieties from Spain: PD = *Pedrosillano*; BL = *Blanco Lechoso*; CA = *Castellano*.

**Table 4:** Sensory analysis of the several ingredients cooked under different conditions, freeze-dried, and rehydrated.

Ingredients Cooked Together for Broth Obtention	Sensory Analysis *	Open Cooker, 100 °C			Pressure Cooker		
		t (min)			t (min)		
		60	90	180	30	60	90
**Chicken (quarters)**	**Sum of ranks**	9b	19ab	26a	12b	19ab	23a
	**Descriptive after cooking**	Tough texture. Poor development of taste and aroma	Cohesive and maintains integrity. Not juicy. Mild taste and aroma but pleasant	Texture: soft and juicy. Chicken broth flavor. Pleasant aroma and appearance	Texture: tough. Mild flavor and aroma to chicken broth. Pleasant appearance	Texture: soft but compact and mildly dry. Pleasant taste and aroma to chicken broth	Texture: soft and disintegrated, lack of cohesion. Pleasant taste and aroma
	**Descriptive after freeze-drying and rehydration**	Texture, flavor and appearance similar to the cooked product	Texture: compact and dry. Taste and aroma similar to the cooked product	Texture: soft and juicy. Flavor, aroma and appearance similar to the cooked product	Texture, flavor and appearance similar to the cooked product	Texture: compact and dry. Taste and aroma: similar to the cooked product	Texture: soft and disintegrated, lack of cohesion. Meat crumbled during mastication. Flavor and appearance similar to the cooked product
**Beef: flank and shank cuts**	**Sum of ranks**	9b	18ab	27a	10b	17ab	27a
	**Descriptive after cooking**	Excessively tough meat, hard to chew. Poor flavor development, very slightly umami. Pleasant appearance	Tough meat. Pleasant taste, aroma and appearance, slightly meaty, umami	Doneness desired. Soft and juicy meat. Intense sapid and aromatic development, very pleasant, umami and meaty touches	Hard, compact meat. Little but pleasant sapid and aromatic development	Tender meat. Pleasant taste, smell and appearance. Soft sapid and aromatic development	Doneness desired. Very soft meat. Soft sapid and aromatic development
	**Descriptive after freeze-drying and rehydration**	Tough and leathery meat, hard to chew. Nice appearance. Taste and aroma similar to the cooked product	Hard, compact meat. Nice look. Little sapid and aromatic development. Pleasant	Maintains structure. Smooth and juicy meat. Very pleasant tasty and aromatic development, similar to the cooked product	Texture, flavor and appearance similar to the cooked product	Texture, flavor, aroma and appearance similar to the cooked product	Doneness desired. Soft and juicy meat. Disintegrates easily, frayed. Good flavorful and aromatic development
**Turnip (*Brassica napus*), Carrot (*Daucus carota sativus*), Leek (*Allium ampeloprasum var. porrum*) and Potato (*Solanum tuberosum*)**	**Sum of ranks**	9b	19ab	26a	9b	18ab	27a
	**Descriptive after cooking**	Texture: tough. Pleasant appearance, flavor and aroma	Smooth, somewhat rough texture. Pleasant taste, smell and appearance	Texture: soft and juicy. Pleasant flavor, aroma and appearance	Hard texture. Little sapid and aromatic development. Pleasing appearance	Slightly rough and fibrous texture. Mild flavor and aroma. Pleasing appearance	Smooth texture. Good flavor and aromatic development. Pleasing appearance
	**Descriptive after freeze-drying and rehydration**	Dry texture and appearance similar to the cooked product	Rubbery texture, but juicy. Flavor and aroma characteristics very similar to those of the cooked product	Maintains structure. Soft and juicy. Maintains flavor and aroma	Texture, flavor and appearance similar to the cooked product	Fibrous, juicy texture. Similar flavor and aroma to the freshly cooked product	Maintains tissue integrity. Soft and juicy texture. Taste and aroma similar to the freshly cooked product
**Broth**	**Sum of ranks**	9b	18ab	27a	9b	20a	25a
	**Descriptive after cooking**	Little sapid and aromatic development. Light umami and meaty flavor	Pleasant sapid and aromatic development, but mild umami and meaty flavor. Aroma and taste associated with plain beef broth	Very nice sapid development. Meaty, tasty, similar to the traditional product. Dense	Little sapid and aromatic development. Light umami and meaty flavor	Pleasant sapid and aromatic development, but mild umami and meaty flavor. Aroma and taste associated with plain beef broth	Meaty, tasty, similar to the traditional product. Dense. Certain "overheated" aftertaste
	**Descriptive after freeze-drying and rehydration**	N/A	Maintains the sapid and aromatic characteristics of the cooked product	Maintains the sapid and aromatic characteristics of the cooked product	N/A	Maintains the sapid and aromatic flavor and aroma characteristics of the cooked product	Maintains the sapid and aromatic characteristics of the cooked product
**Ingredients cooked independently**		**Open Cooker, 100 °C**			**Pressure Cooker**		
		**t (min)**			**t (min)**		
		**15**	**30**	**60**	**15**	**30**	**60**
**Cabbage (*Brassica Oleracea*)**	**Sum of ranks**	10b	26a	18ab	25a	18ab	11b
	**Descriptive after cooking**	Texture: tough. Flavor and aroma: poorly developed. Pleasant appearance	Smooth texture. Nice taste and appearance	Pleasant flavor and appearance but soft texture	Smooth texture. Maintains integrity. Nice taste and appearance	Texture: disintegrated, extremely soft	Texture: disintegrated, extremely soft
	**Descriptive after freeze-drying and rehydration**	Similar texture and flavor to the cooked product	Maintains integrity. Similar flavor and aroma to the cooked product	Maintains integrity. Similar flavor and aroma to the cooked product	Similar flavor and aroma to the cooked product	N/A	N/A
**Spicy pork sausage (Spanish *chorizo*)**	**Sum of ranks**	27a	17ab	10b			
	**Descriptive after cooking**	Texture: compact, soft and juicy. Appearance, flavor and aroma typic of the product	Texture: compact and juicy. Typic (characteristic) flavor and aroma of the product	Compact but dry texture. Characteristic flavor of the product but with aftertastes with slight touches of rancid. Nice appearance			
	**Descriptive after freeze-drying and rehydration**	Soft texture but fatty. Characteristic flavor and aroma of the product, with rancid nuances. Nice appearance	Texture: compact and dry. flavor and aroma: similar to the cooked product	Texture: compact and dry. flavor and aroma: similar to the cooked product			
**Pork blood sausage (Spanish *morcilla*)**	**Sum of ranks**	27a	15ab	12b			
	**Descriptive after cooking**	Texture: compact, soft and juicy. Appearance, flavor and aroma typic of the product	Texture: disintegrated, lack of cohesion. Appearance, flavor and aroma typic of the product	Texture: dry and disintegrated, lack of cohesion. Appearance, flavor and aroma typic of the product but with slight hints of rancidity			
	**Descriptive after freeze-drying and rehydration**	Texture: soft. Pleasant appearance, flavor and aroma; typic of the product	Texture: compact and dry. flavor and aroma: similar to the cooked product	Texture: compact and dry. flavor and aroma: similar to the cooked product			

* The sum of ranks corresponds to the analysis of the freshly cooked samples. The same ranking was obtained after freeze-drying and subsequent rehydration. Values without a common letter within the same row for the same cooking system differ significantly (*p* < 0.05).

## Data Availability

All related data and methods are presented in this paper. Additional inquiries should be addressed to the corresponding author.
